# Agreement between mechanical and digital skinfold callipers

**DOI:** 10.1177/02601060221119247

**Published:** 2022-08-15

**Authors:** Ana Catarina Vaz Pinheiro de Furtado Faria, Diogo Vicente Martinho, Bruno Rafael Ribeiro Abreu, Bruno Rafael Costa Franco, Lara Alexandre Moreira Carrilho, Madalena Carraça Azaruja, Pedro Miguel Tavares Mendes, Mariana Duarte Simões Serra, João Alexandre Teixeira Lemos, João Paulo de Figueiredo

**Affiliations:** 1 Laboratory for Applied Health Research (LabinSaúde), Coimbra, Portugal; 2 Polytechnic of Coimbra, Coimbra Health School, Dietetics and Nutrition, Coimbra, Portugal; 3 Faculty of Sports Science and Physical Education, 37829 University of Coimbra, Coimbra, Portugal; 4 Sporting Clube de Portugal, Lisbon, Portugal; 5 Clinic Studio Medicina & Saúde, Águeda, Aveiro, Portugal; 6 Futebol Clube de Paços de Ferreira, Paços de Ferreira, Portugal; 7 Department of Complementary Sciences, School of Health Technology of Coimbra, Coimbra, Portugal

**Keywords:** adipose tissue, clinical examination, body composition, anthropometry, skinfold thickness

## Abstract

**Background:** Skinfold callipers are often used in clinical practice to estimate subcutaneous adipose tissue thickness. Recently, LipoTool emerged as a potential digital system to measure skinfolds, however comparisons with competing equipment are lacking. **Aim:** The aim of this study was to test the agreement between two competing skinfold callipers (digital and mechanical). **Methods:** The sample included 22 healthy male adult participants. A certified observer measured eight skinfolds twice using different skinfold callipers (digital and mechanical). Differences between equipment were tested using Wilcoxon signed rank test The distribution of error was examined using the normality test **Results:** Differences between skinfold callipers were significantly in five skinfolds: triceps (Z  =  -3.546; P < 0.001), subscapular (Z  =  -3.984; P < 0.001), suprailiac (Z  =  3.024; P  =  0.002), supraspinale (Z  =  3.885; P < 0.001), abdominal (Z z = −2.937; P  =  0.003), thigh (Z  =  -2.224; P  =  0.026) and calf (Z  =  -2.052; P  =  0.040). Differences between callipers were constant. **Conclusions:** Mechanical and digital callipers tended to record different values of skinfold thickness. Clinical examination should consider equipment-related variation in fat mass estimation.

## Introduction

The study of human body composition includes different methodologies to examine the compartments of body mass (Wang et al., 1992). Given the negative relationship with metabolic parameters, fat mass (FM) estimation has potential relevance for clinics and nutritionists (Madden and Smith, 2016). Although dual-energy X-ray absorptiometry (DXA) and bioelectrical impedance analysis are widely used to estimate FM, these technologies require specialized equipment (Ward, 2018). In parallel, sex-specific equations based on skinfolds to predict body density and, consequently FM, were derived from hydrostatic weighing (Jackson and Pollock, 1985). Subcutaneous adipose tissue thickness was measured using a skinfold calliper (Jackson and Pollock, 1985). The equipment is portable, associated-protocols are non-invasive and consequently, skinfold callipers are often used as the most common instrument to estimate fatness (Madden and Smith, 2016).

Different type of callipers has been used in clinical practice. Note, however, protocols require standardized procedures and training in order to reduce intra and inter-individual variability measurement (Lohman et al., 1988). The instructions of international references called for the use of specific callipers which should exert a constant pressure in subcutaneous adipose tissue (Eston and Reilly, 2009). More recently, sophisticated callipers emerged as potential instruments to estimate subcutaneous adipose tissue thickness (Quintas et al., 2015; Tafeit et al., 2015). For example, a digital skinfold calliper (Adipsmeter) maintained a constant pressure of 10 gf/mm^2^ between tips while in traditional callipers pressure tended to decrease with the opening of jaw. The Adipsmeter is based on a Harpenden calliper, electronically adapted and incorporated a digital sensing system (LipoTool) reducing the intra-observer variability (Quintas et al., 2015). Among 49 participants, differences between estimated %FM obtained by a traditional calliper and LipoTool were considerable at higher values of fatness (Restivo et al., 2012). Comparisons between competing equipment were limited to body density estimations and did not contrast specific skinfold sites (Restivo et al., 2012). Age and sex-specific equations are systematically used by clinics to evaluate %FM. Meantime, disagreement between callipers may affects the interpretation of this parameter which in turns have impact on nutritional status and interventions.

Given the described differences between two concurrent techniques, the aim of the present study was to examine the quantitative agreement in specific skinfold sites using two different callipers (mechanical and digital) assessed by a certified observer. Since traditional callipers do not maintain a constant pressure, particularly in participants with superior values of FM, it was hypothesized differences between concurrent equipment.

## Methods

### Procedures and ethical requirements

The present study was approved by the Ethics Committee in the Institute Polytechnic of Coimbra (N.°92_CEIPC/2021; approval at 8^th^ June 2021) and followed the recommendations from the Declaration of Helsinki produced by the World Medical Association for research with humans. Participants were previously informed about the nature, aim, risks of the study and, subsequently, provided written informed consent.

### Participants

The sample included 22 healthy male Caucasian participants aged 19–30 years. Inclusion criteria were: (i) participants were physically active; (ii) body mass index < 30 kg.m^−2^.

### Anthropometry

**Figure 1.**
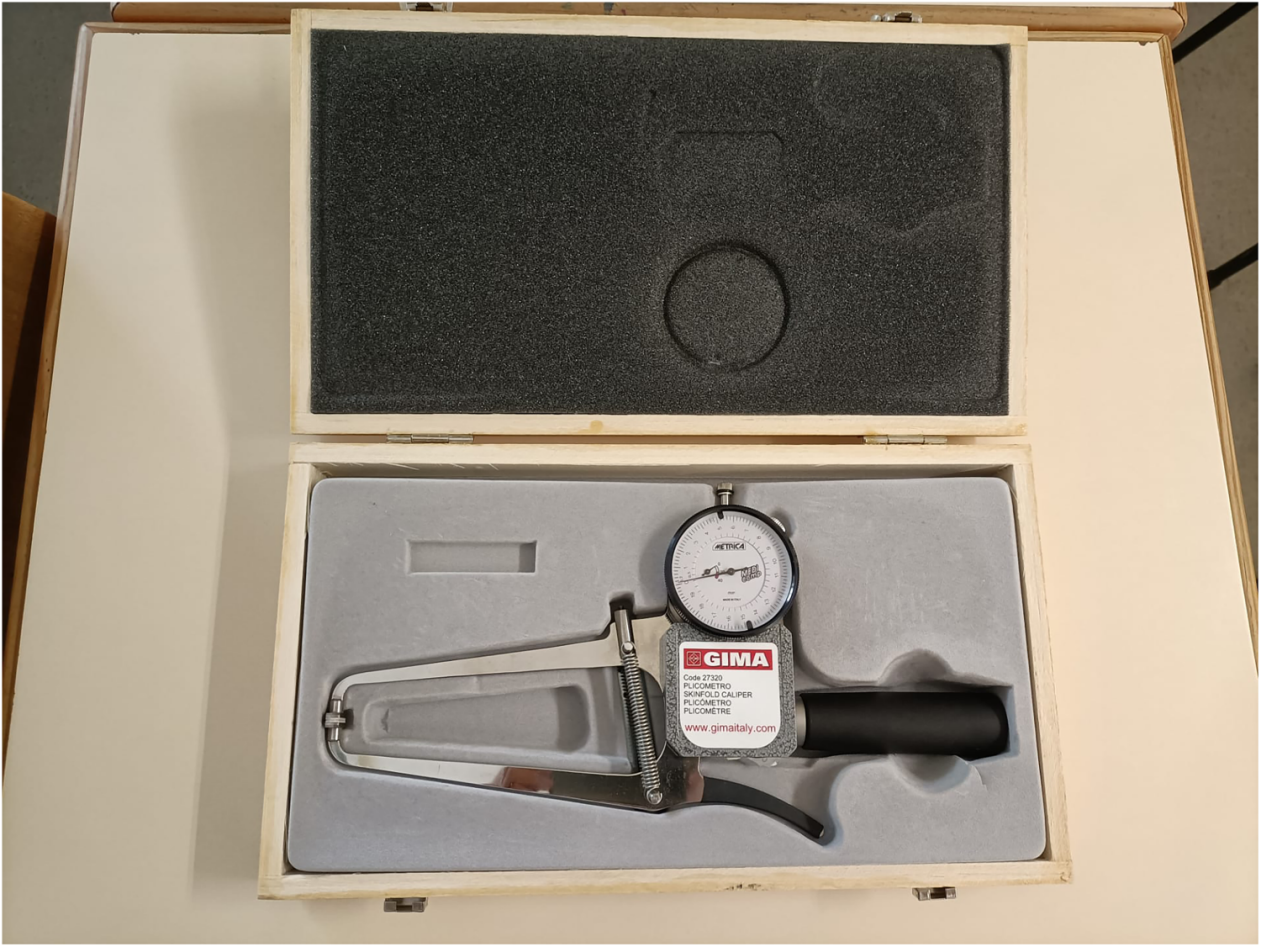
Mechanical calliper.

**Figure 2.**
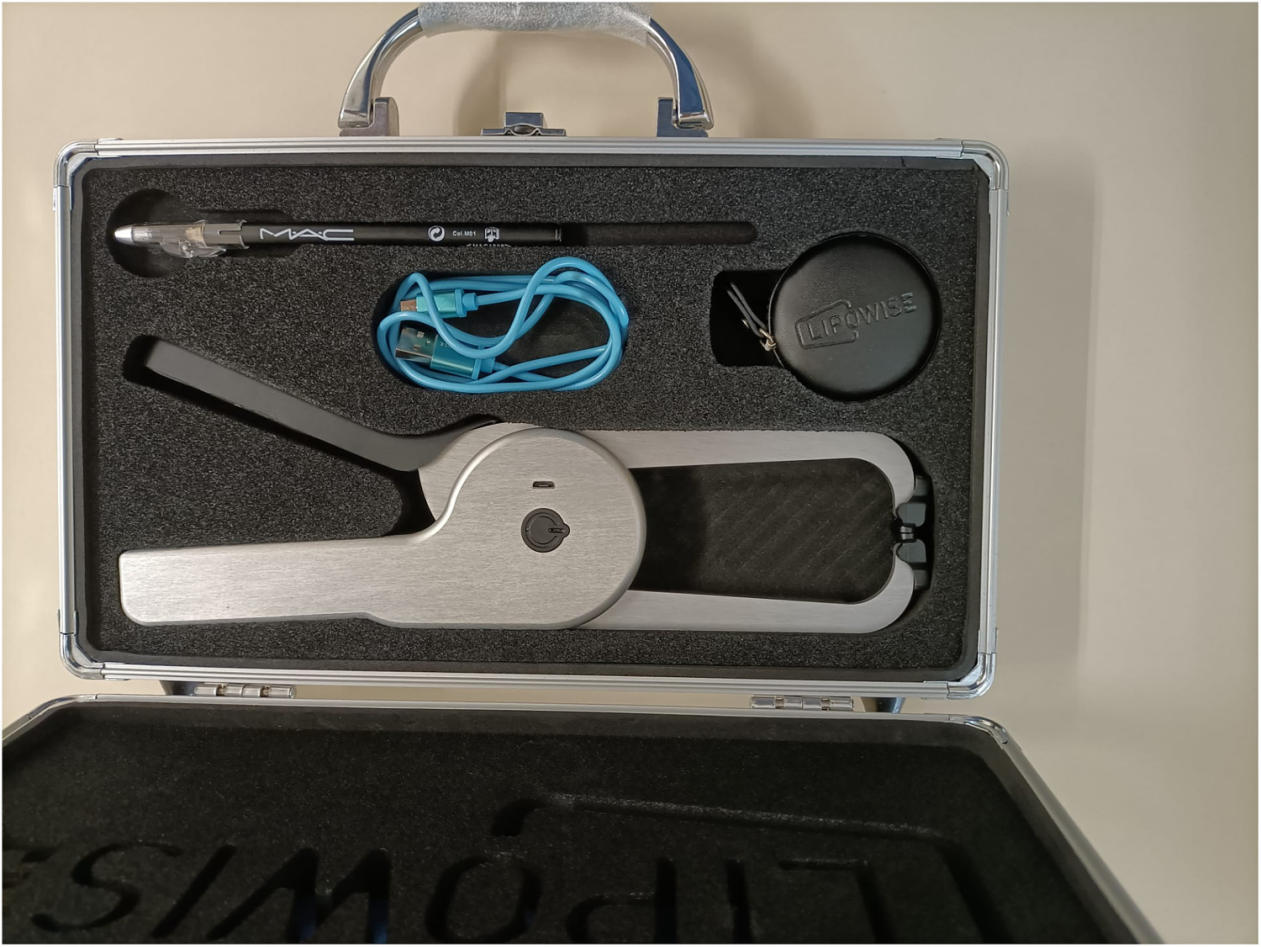
Digital calliper.

Skinfold thickness was measured at eight sites (triceps, subscapular, biceps, suprailiac, supraspinale, abdominal, thigh and calf) to the nearest 0.1 mm by certified observer and following the protocol recommended by International Society for the Advancement of Kinanthropometry (ISAK). Two different skinfold callipers were used: mechanical (see Figure 1) and digital (see Figure 2). The latter included an integrated system that also incorporates an antenna AirPCOn interfaced with a computer and a software application. Two measurements using different equipment were obtained individually for each skinfold. The mean of observations was retained for analysis.

### Statistical analysis

Normality of distribution was checked with Shapiro-Wilk. Given the non-normality of distribution differences between two equipment were compared based Wilcoxon signed-rank test. Descriptive statistics (mean and standard deviation) for skinfolds were calculated. In addition, Bland-Altman plots and limits of agreement were calculated to visualize absolute differences and respective mean (Bland and Altman, 1986). Linear regressions were applied separately for each skinfold and sum of skinfolds. Afterwards, Shapiro-Wilk test was used to examine the distribution of residuals (i.e. normality of error) considering the two instruments.

## Results

**Figure 3.**
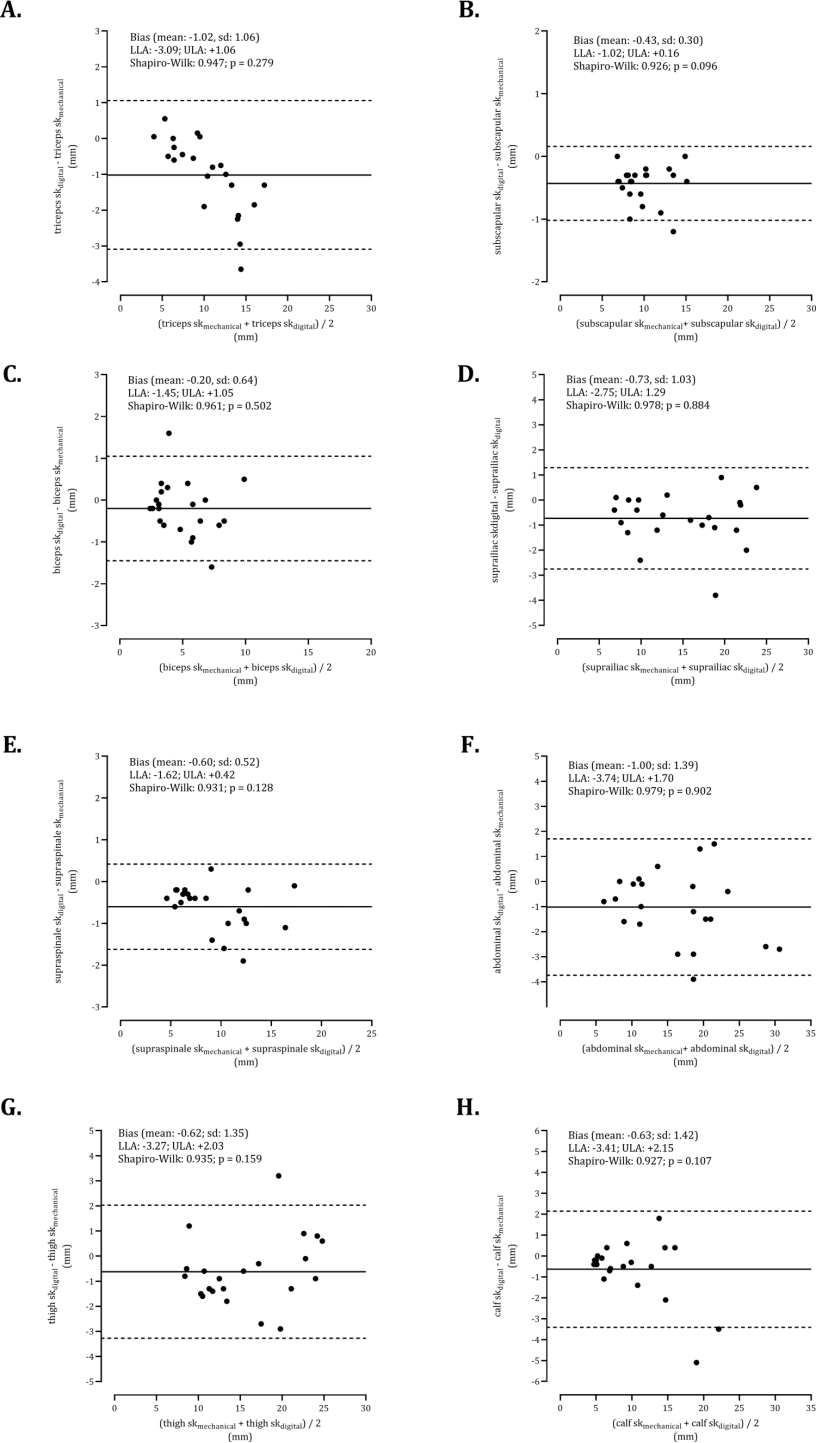
Agreement of digital and mechanical callipers for triceps (panel a), subscapular (panel b), biceps (panel c), suprailiac (panel d), supraspinale (panel e), abdominal (panel f), thigh (panel g) and calf (panel h) skinfolds.

**Table 1** summarizes the comparisons of two callipers (mechanical and digital). Significant intra-individual variability was noted in triceps (z = -3.546; P <0.001), subscapular (z = -3.984; P <0.001), suprailiac (z = -3.024; P = 0.002), supraspinale (z = -3.885; P <0.001), abdominal (z = -2.937; P <0.003), thigh (z = -2.224; P = 0.026), calf (z = -2.052; P = 0.040) skinfolds. Consequently, the sum of skinfolds obtained by mechanical adipometer was significantly higher than in digital calliper (z = -4.107; P <0.001). As illustrated in **Figure 3**, skinfolds assessed by digital calliper were, on average, systematically less than mechanical calliper. The normality test of residuals showed that differences between equipment did not tend to increase with the respective mean of skinfolds.

**Table 1.** Means and standard deviations for mechanical and digital callipers, z-score and significance value extracted from Wilcoxon signed-rank test.

Dependent variable*	type of skinfold calliper (mean ± standard deviation)		Wilcoxon-test	
	mechanical	digital	z-score	p
Triceps skinfold	10.9 ± 4.2	9.9 ± 3.4	−3.546	<0.001
Subscapular skinfold	10.1 ± 2.6	9.7 ± 2.6	−3.984	<0.001
Biceps skinfold	5.0 ± 2.2	4.8 ± 2.1	−1.836	0.066
Suprailiac skinfold	15.1 ± 5.9	14.4 ± 5.8	−3.024	0.002
Supraspinale skinfold	9.5 ± 3.7	8.9 ± 3.5	−3.885	<0.001
Abdominal skinfold	16.6 ± 6.9	15.6 ± 6.5	−2.937	0.003
Thigh skinfold	16.1 ± 5.5	15.5 ± 5.5	−2.224	0.026
Calf skinfold	10.0 ± 5.5	9.3 ± 4.8	−2.052	0.040
Sum of skinfolds	93.4 ± 30.5	88.3 ± 28.7	−4.107	<0.001

*Dependent variables were expressed in millimetres (mm).

## Discussion

The aim of this study was to test the concordance between competing skinfold callipers. Adipsmeter which is part of an integrative system tended to produce systematically less values than mechanical calliper. Additionally, the agreement between callipers showed that the error was constant. Comparisons between different type of skinfold callipers were reported in studies of youth (Tafeit et al., 2015), adult and elderly (Restivo et al., 2012). For example, among 371 Estonian males aged 9–13 years, skinfolds measured using a plastic calliper were systematically higher in comparison to a lipometer. Meantime, the agreement between calipers decreased above 10 mm and thigh emerged as the most problematic skinfold (Tafeit et al., 2015). In the present study, differences between concurrent equipment were constant. The contrasted results across studies may reflect sampling, age variation or callipers used. The sample of this study considered healthy adult participants while, the Estonian sample involved pre-pubertal and adolescent males.

A previous study examined the agreement between estimated %FM using LipoTool (a similar calliper was used in the current study) and Harpenden callipers (Restivo et al., 2012). Among male and female participants, Harpenden calliper tended to produce systematically lower values than LipoTool which is consistent with the findings of present study. Interestingly, differences between equipment were considerable at higher values of %BF. These results may be explained by the differences between callipers. Nevertheless, the digital calliper has a unique characteristic – a transmission rate of 60 samples per second which in turns is related with the constant tissue compressibility during skinfold measurement (Quintas et al., 2015).

Differences between the mechanical and digital callipers were noted on the skinfolds examined in the present study. However, particular skinfolds emerged as relevant to estimate fat mass. The impact of lower limb skinfolds was previously assessed in a study with 21 male adults. Overall, thigh skinfold explained 79% of %FM assessed by dual energy x-ray absorptiometry (Eston et al., 2005). Additionally, triceps, suprailiac and abdominal skinfolds were included to predict body density (Jackson and Pollock, 1985). Therefore, associated-variation in skinfold callipers measurements should be considered when different studies are compared on estimated body composition.

The present study compared skinfold measurements using two type of skinfold callipers and did not test the concordance between equations. Future studies should contrast FM estimated by anthropometric equations (using the LipoTool) with more sophisticated methods of measuring FM or fat tissue (i.e. ultrasound imaging, air displacement plethysmography or DXA). In summary, assessment of subcutaneous adipose tissue using competing skinfold callipers showed different estimations in specific sites. This study highlighted the differences between equipment for measuring fatness with potential implications during clinical assessment.
